# Essence of Disease

**Published:** 1869-02

**Authors:** 


### HALL'S JOURNAL OF HEALTH.

VOL. XVI. FEBRUARY, 1869. No. 2.

### ESSENCE OF DISEASE.

THE science of medication, as far as it has become a science, is beautifully simple, and carries with it, to the thoughtful and logical mind, a high degree of interest, which the reader may presently see.

All disease may be said to be founded in an unequal distribution of the blood, while its equilibrium is essential to high health and manly vigor.

While it is true that too much blood at a particular part of the body, causes a diseased condition of that part, such as head-ache, if in the head; the same amount of blood may give two very different diseases, or two very different symptoms or manifestations, according to the set of vessels which contains that excess of blood, whether artery or vein.

Many know the difference between a dull, heavy, depressive head-ache which invites repose, and the sharp, piercing pain which makes sleep an impossibility; between the burning feet in some forms of dyspepsia, which makes standing on the snow a perfect luxury, and the cold, clammy sweat of cholera consumption.

The blood is distributed to the body through the veins and arteries, and where there is an artery, there must be a vein. The blood flows through the veins like a slow, steady river; but through the arteries like the dash of the leaping waters.

When there is too much blood in the veins, it is called "Congestion," because it packs, it gorges, it dams up; when there is too much in the arteries, it is called "Inflammation," because it fires up the parts, makes them hot, red, flame-like.

When the veins of a part are too full, there is a dull pain, and the color is inclined to a black red; when the arteries are too full, there is a fierce, quick darting pain, and a fiery appearance.

Disease being a breaking up of the equilibrium of the blood, whatever has a tendency to restore that equilibrium, to withdraw the blood from the over-stocked part, promotes health to that extent.

Although the very last part to die, death, in a sense, begins at the heart, by its not being able to relieve itself at a given beat, of all the blood that is in it; the next beat, and there is a greater surplus, and with that, less power to distribute the vital fluid to the extremities of fingers, feet and skin; then they begin to grow clammy and cold, and death-like. But if, almost in the article of death, any great physical or mental shock can be imparted, by which the heart shall bound with a superhuman throb, and clear itself of its entire contents, life is saved.

The devoted and indefatigable missionary Durfee, was dying of low fever; the cold extremities, the fixed eye, the labored breathing, all showed that the powers of life were rapidly wasting away, although a loud voice would arouse him to consciousness; this suggested to the physician that if the heart could be relieved of its load of blood, if the equilibrium of the circulation could be for a moment restored, he might be saved. He was placed on the floor, and buckets of water were poured upon the body from the height of a man. He seemed to wake up as from a heavy sleep or dream; the circulation was re-established, natural warmth restored, the voice became as clear and the mind as active as in health; he fondled his youngest child, and for a while all seemed hopeful, but nature had lost her recuperative power, had not strength to sustain herself, and he gradually pined away.

A poor old woman had been bed-ridden for years with rheumatism, when being left alone one day, she waked up to find the house on fire; with one bound she leaped from her couch, ran as fast as any body, and thereafter could walk as well as others of her age.

It is related of a celebrated physician, that journeying one day, he heard that a lady was dying with a low fever, and greatly desired to see him, as they had not met since childhood, when they were very dear friends. On the instant of entering the chamber, he clapped his hands joyously, and exclaimed, "The Eagle's Nest," and she lived. They had spent many happy hours of school-time around the eagle's nest, and all the associations coming back upon her in an instant, caused a shock which other means were powerless to produce.

Within a short time, a young man named Joseph Wheeler, of New Orleans, who had been deaf and dumb for four years, in consequence of some sickness, sauntered up to a cannon's mouth, without any one noticing it; the match was applied, when it was too late to snatch him away. He fell down as if dead, but presently came to himself, speaking as fluently as he ever did, and answering all questions put to him, to the great wonderment of the by-standers.

All are familiar with the palor of the face induced by sudden alarm or other great excitement; it is because that under the influence of great mental or physical shocks, the blood retreats to the heart in extra quantities, draining the other portions of the body, leaving such of them as were diseased by reason of their having too much blood there, in their natural or more healthful condition.

While the first effect of a shock is to send the blood of the body in upon the heart, the second effect is for the heart, by the excess of stimulus, to make a desperate effort to relieve itself; this is "reaction," but in making that clearance, although it received more blood from the diseased part than naturally belonged to it, it sends back only its proper proportion to that part, hence the restoration of the equilibrium and return to health.

In the first case, the excess of blood, or obstruction, was in the head, hence the stupor; in the old woman, it was in the joints; in the young man, it was in the ear; while in the case of the "Eagle's Nest," it was in the internal organs, the liver, most.

But there are less heroic methods of restoring the equilibrium; more quiet ways of equalizing the circulation.

Persons have appeared to be dying when the mustard or blister plaster applied to the wrists and ankles has drawn the blood to the parts, evidenced by their being reddened, thus relieving the heart and saving life.

A man sits down to dinner with a severe head-ache, eats heartily, and feels it no longer. It is because an excess of blood is required in the stomach when it is filled with food; the brain, by furnishing its quota, is relieved of the surplus blood which caused the pain, and the equilibrium is restored. But a hearty meal will not always remove head-ache, for reasons not necessary now to be explained.

Insane persons cannot sleep enough, the arteries of the brain are too full of blood, it is sent to them in too large quantities; hence, in some cases, sleep has been obtained by feeding the lunatic six or eight times a day, thereby keeping the stomach full of food, and drawing the blood there for its digestion, thus relieving the brain. The medical proprietor of a lunatic asylum in England has pursued a plan of this sort for fifty years with very successful results. Most observant readers have felt the somnific effects of a hearty dinner.

It is by restoring the equilibrium of the circulation, that the "reaction" of the cold shower-bath removes some forms of disease, which failed to be reached in other ways.

The practical lesson of this article is, they will live the healthiest and the longest, who have the equilibrium of the circulation least interfered with; hence an important means of avoiding sickness and attaining a good old age, is to live quietly, uniformily and regularly; there is no preventive of disease equal to this, and it is well worth while for all to practice it.

